# A case of Hyperkalemia-induced Brugada Phenocopy: a rare but serious electrocardiographic imposter

**DOI:** 10.1093/omcr/omaf036

**Published:** 2025-05-28

**Authors:** Abraam Rezkalla, Islam Rajab, Fathi Milhem, Moath Hattab, Yezin Shamoon, Fayez Shamoon

**Affiliations:** St. Joseph’s University Medical Center, Department of Internal Medicine, Paterson, NJ 07470, United States; St. Joseph’s University Medical Center, Department of Internal Medicine, Paterson, NJ 07470, United States; Department of Medicine, An Najah National University, Nablus, Palestine; Department of Medicine, An Najah National University, Nablus, Palestine; St. Joseph’s University Medical Center, Department of Cardiology, Paterson, NJ 07470, United States; St. Joseph’s University Medical Center, Department of Cardiology, Paterson, NJ 07470, United States

**Keywords:** Brugada syndrome, Brugada Phenocopy, Hyperkalemia, sudden cardiac death, channelopathy

## Abstract

Brugada Syndrome is a potentially fatal, hereditary cardiac disorder that may precipitate sudden cardiac death if not identified. An electrocardiogram (ECG) revealing a characteristic coved ST segment elevation followed by negative T wave in a right-sided precordial lead is pathognomonic for the Brugada pattern. Recently it has been shown that certain abnormalities, such as an electrolyte disturbance, may precipitate a Brugada pattern on ECG. Once the precipitating factor is treated, the rhythm reverts to baseline, coining the term Brugada Phenocopy. Here we present a captivating case of a 40-year-old female who was found unresponsive and determined to have a Brugada pattern on ECG secondary to hyperkalemia. Once appropriately treated, ECG reverts to normal baseline.

## Introduction

Brugada Syndrome is a hereditary cardiac condition, responsible for over half of sudden cardiac deaths in young individuals. It is characterized by distinct electrocardiogram (ECG) patterns, particularly in the right precordial leads [1]. The term Brugada Phenocopy has been introduced, referring to ECG patterns that resemble Brugada Syndrome but are induced by external factors such as hyperkalemia, mechanical pressure, or myocardial disorders [2]. Distinguishing between Brugada Syndrome and Brugada Phenocopy is vital, as misdiagnosis may lead to improper treatment, especially in critical cases.

## Case presentation

A 40-year-old female presented to the Emergency Department after being found unresponsive by her son with questionable vomit around her mouth. The patient was last seen awake and alert 12 hours prior.

## Investigation

Upon arrival at the Emergency Department, the patient was found to be lethargic and unable to follow commands; however, she would open her eyes spontaneously. The patient was hypotensive with a blood pressure of 66/47 mmHg, tachycardic to 124 beats per minute, had a respiratory rate of 19 breaths per minute, and an Oxygen Saturation of 82% on room air oxygen. The patient was escalated to Non-Rebreather Mask on 100% FiO2 (Fraction of inspired oxygen). An Arterial Blood Gas from the radial artery was obtained while on a Non-Rebreather Mask on 100% FiO2 revealing a pH of 7.24, a Partial Pressure of Carbon Dioxide of 46 mmHg, a Partial Pressure of Oxygen of 37 mmHg, and a Bicarbonate level of 19.7 mmol/l. Findings consistent with metabolic and respiratory acidosis, a complete metabolic panel showed a significantly elevated level of Potassium at 6.7 mmol/l, also consistent with the metabolic acidotic state of the patient, which we contribute to many causes, first of all the prolonged immobilization, which leads to rhabdomyolysis, which contributes to the acidotic state, leading to extracellular shift of potassium, along with the hypotensive and hypoxic state leading to impaired renal function and increased tissue breakdown respectively.

**Figure 1:**
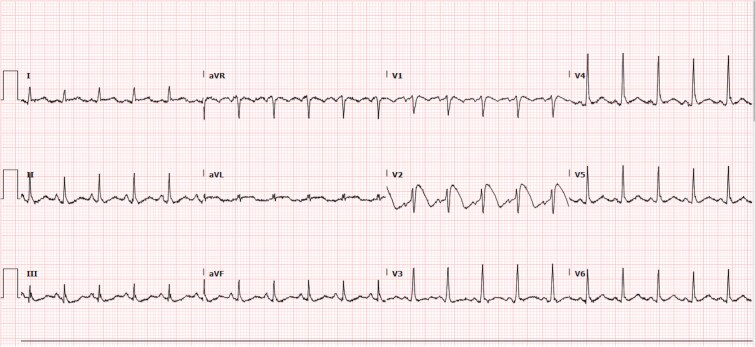
Initial ECG: Sinus rhythm with Brugada type 1 pattern in lead V2: A coved-type ST segment elevation followed by an inverted T wave, in leads V3-V5 there’s peaked T waves, consistent with hyperkalemia. PR interval: 119 ms.

The patient was later intubated and placed on mechanical ventilation due to bradypnea and for airway protection due to decreased consciousness. Upon obtaining diagnostic imaging, an initial Electrocardiogram (ECG) was significant Brugada type 1 pattern in lead V2, by a coved-type ST segment elevation followed by an inverted T wave, in leads V3-V5, there are peaked T waves, consistent with hyperkalemia. (Fig. 1) Upon further chart review and obtaining information from the next of kin, the patient did not have a reported history of syncope or cardiac arrest as well as no family history of sudden cardiac death. Previously recorded ECGs were also noted to be normal with the patient in normal sinus rhythm, with no ST segment elevations or depressions and no T wave changes.

## Differential diagnosis

The patient was admitted to the Medical Intensive Care Unit requiring mechanical ventilation and vasopressors under the diagnosis of Acute Hypoxic Respiratory Failure as well as a possible Brugada pattern on ECG secondary to Hyperkalemia.

## Treatment

**Figure 2:**
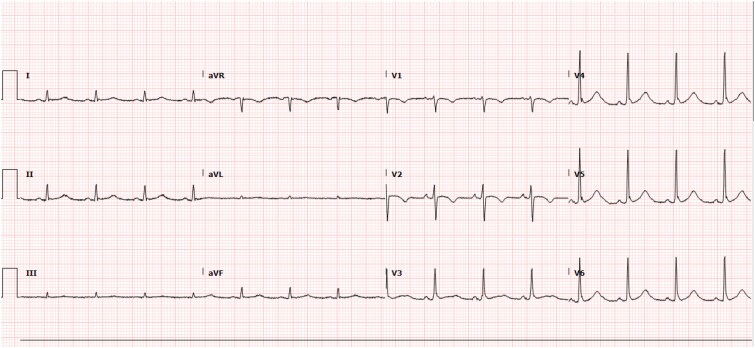
Type-1 pattern diminished in lead V2 revealing normal sinus rhythm. PR interval: 133 ms.

The patient was started on a continuous infusion of Normal Saline running at a rate of 100 ml/Hr and was given Calcium Gluconate 1000 mg Intravenously and 10 Units of 0.1 mL Regular Insulin to rapidly treat the hyperkalemia. Subsequent potassium levels were noted to be 5.1 mmol/l. Serial ECGs were performed and once potassium levels normalized, an ECG revealed normal sinus rhythm with a T wave inversion in lead II; however, the previously recorded ST elevation in lead V2 with biphasic T wave changes had since improved. The patient remained in the Medical Intensive Care Unit until stabilization with eventual extubation and recovery. (Fig. 2). With consent, the attached ECGs demonstrate the rapidly improved patterns.

**Figure 3:**
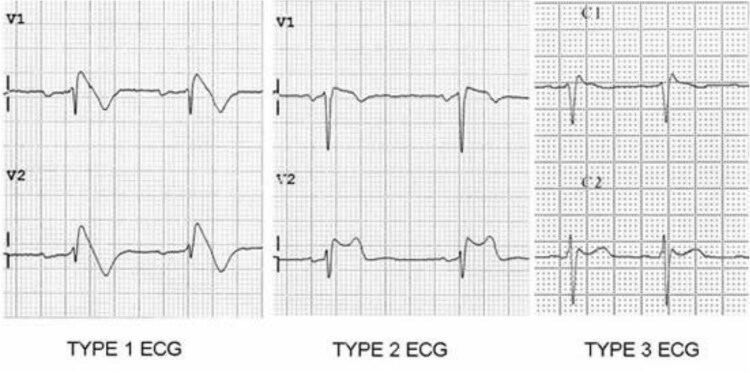
Type 1 is characterized by a prominent coved ST-segment elevation with a J-point amplitude of ≥2 mm with a negative T wave. Type 2 with a ≥ 2 mm J-point elevation, ≥1 mm ST-segment elevation, and saddled appearance with a biphasic or positive T wave following. Type 3 can have either saddled, coved appearance, or both however with an ST-segment elevation < 1 mm. While type 1 is diagnostic, either latter are only suggestive. [12].

## Discussion

Brugada Syndrome is considered one of the few familial sudden cardiac death syndromes usually associated with a structurally normal heart. Generally attributed to over 50% of all sudden cardiac deaths in young individuals, Brugada Syndrome is an extremely significant diagnosis to identify as it can lead to early identification in family members [3].

There are three recognizable repolarization patterns associated with the Brugada pattern when found in the right-sided precordial leads on an ECG (Fig. 3). The first being the Type 1 pattern which displays a high initial take-off of the ST-segment elevation that is greater or equal to 2 mm with a sudden down-sloping concave or rectilinear contour followed by a negative symmetric T-wave. The second being the Type 2 pattern defined as a high initial take-off (r’) greater or equal to 2 mm from the isoelectric baseline that is convex, which is dissimilar to type 1, followed by a carriable T wave in lead V1 and semi-variable T wave in lead V2 [4]. The third has a saddleback, coved type, or even both, however, with the < 1 mm elevation in the ST-segment. While type 1 is strictly the diagnostic pattern, the other two patterns are suggestive of the disease. To be diagnostic, the ECG pattern must be associated with one of the following clinical criteria to make the diagnosis: documented ventricular fibrillation or polymorphic ventricular tachycardia, a family history of sudden cardiac death, syncope, or inducibility of VT with programmed electrical stimulation [5].

While genetic mutations that lead to QT prolongations due to ion channel dysfunctions were being discovered, similar analogies were substantiated in Brugada Syndrome cases. A hereditary pattern was suspected in only half of Brugada cases. Given that there was no suspected structural morphology, it could demonstrate an argument for it to be a type of channelopathy allowing it to be similar to others [6].

A relatively new diagnosis, termed Brugada Phenocopy by Baranchuk et al., is attributed to a Brugada ECG configuration without the clinical indication of Brugada Syndrome, which if misdiagnosed can lead to fatal ventricular tachyarrhythmia [2, 4, 7].

Riera et al. have recently defined the term Brugada Phenocopy as ‘an environmental condition that imitates one produced by a gene.’ Multiple extra somatic factors that have appeared to mimic such a pattern causing Brugada Phenocopy [BrP], include but are not limited to, electrolyte abnormalities, mechanical compression of the right ventricle, myo- and pericardial diseases in addition to rhabdomyolysis [8]. Hyperkalemia to be specific has proven to produce a Brugada Phenocopy pattern due to ionic gradient aberrations. Common ECG changes secondary to hyperkalemia include ‘tenting’ or ‘peaking’ of T-waves in mild hyperkalemia and eventual loss of T-wave regularity leading to a sinus-wave pattern in more severe concentrations [4]. At a cellular level, phase 0 of the myocardial action potential is altered leading to slower conduction velocity and subsequent widening of the QRS complex. Hyperkalemia can also lead to the Brugada type-1’s distinct ST elevation pattern due to the inactivation of cardiac sodium channels thereby decreasing resting membrane potential. These alterations in by itself cause the transmural myocardial incline and gradual conduction at the right ventricular outflow tract, triggering a Brugada pattern [9].

The Brugada pattern, once noted on an ECG, should prompt a hasty investigation of the true cause. A substantial inquiry while history taking is necessary in identifying key clinical indications to distinguish between Brugada syndrome and Brugada phenocopy. A family history of idiopathic ventricular tachycardia, ventricular fibrillation and/or sudden cardiac death is critical for diagnosis. New methodologies for measuring Brugada ECG patterns have been attempted to differentiate between true syndrome and phenocopy yet have presented a seemingly low sensitivity and specificity as well as positive and negative predictive values providing further confirmation that the two patterns are undistinguishable [10]. Rapidly prompt recognition of this clinical and morphological entity may be lifesaving as treatment of hyperkalemia traditionally will result in a resolution of Brugada ECG Pattern rather than treatment of wide complex rhythms [11].

We provide a case that presented in a severely ill state, but yet showed rapid resolution after treating the underlying cause, hereby emphasizing the role of searching for reversible causes of Type 1 Brugada pattern on ECG, to differentiate Brugada syndrome from phenocopy, as a simple electrolyte panel can be enough for finding the cause and avoiding any unnecessary procedures or interventions.

We also highlight a case of Brugada phenocopy caused by metabolic and respiratory acidosis along with hyperkalemia, possibly attributed to multiple causes, including prolonged immobilization causing rhabdomyolysis, and prerenal acute kidney injury, due to hypotension and hypoperfusion, and the decreased respiratory drive of the patient.

## Conclusion

Since being termed in 2012, Brugada Phenocopy has been characterized in over 100 cases worldwide [12]. Brugada Phenocopy should always be considered when coming across a patient with a Brugada-like pattern on ECG to prevent mismanagement and irrational tests. However, a very diligent history should be taken to rule out the possibility of Brugada Syndrome.

Lastly, our intriguing case reminds the physician to be cautious when seeing such patterns on an electrocardiogram allowing them to treat the acute abnormality accordingly without hesitation.

## Data Availability

Not applicable.
